# A structural perspective on α‐glucan catabolism in oxygenic phototrophs

**DOI:** 10.1111/tpj.70732

**Published:** 2026-02-11

**Authors:** Sofia Doello, Dmitry Shvarev

**Affiliations:** ^1^ Microbiology Department Institute for Integrative Biology of the Cell, CEA, CNRS, Université Paris‐Saclay Gif‐sur‐Yvette France; ^2^ Microbiology and Organismic Interactions, Interfaculty Institute for Microbiology and Infection Medicine University of Tübingen Tübingen Germany; ^3^ Department of Biology/Chemistry, Structural Biology of Photosynthetic Organisms Section University of Osnabrück Osnabrück Germany; ^4^ Center of Cellular Nanoanalytics Osnabrück (CellNanOs) University of Osnabrück Osnabrück Germany

**Keywords:** chloroplast, cryo‐EM, cyanobacteria, glycogen, starch, α‐glucan

## Abstract

Starch and glycogen are the main α‐glucan storage polymers in oxygenic photoautotrophs, ensuring metabolic continuity during day/night cycles and environmental stress. Their mobilization requires a suite of catabolic enzymes whose activities are tightly regulated to balance carbon storage with energy demands. Recent structural studies have provided key mechanistic insights into how these enzymes recognize their substrates, transition between active and inactive states, and respond to cellular signals. In this review, we summarize advances in the structural biology of starch and glycogen degradation in plant chloroplasts and cyanobacteria. We highlight the architectural features that determine substrate specificity and examine how regulatory elements, such as redox switches, proteolytic events, and protein–protein interactions modulate enzyme activity. These structural and mechanistic insights inform how oxygenic phototrophs manage carbon storage to ensure survival under fluctuating environmental conditions.

## Significance Statement

Efficient mobilization of carbon storage polymers is essential for the survival of oxygenic phototrophs under fluctuating environmental conditions. Recent structural studies have uncovered the molecular mechanisms that govern starch and glycogen degradation in photosynthetic organisms. This review integrates these advances to highlight how enzyme architecture and regulation orchestrate carbon mobilization in plant chloroplasts and cyanobacteria.

## INTRODUCTION

α‐Glucans are essential carbon storage polymers found across all domains of life. Composed of glucose units linked by α‐glycosidic bonds, they play a central role in energy homeostasis. Among them, starch and glycogen are the most prevalent α‐glucans used for carbon storage (Reddy Shetty et al., 2021). In photosynthetic organisms, these polymers are synthesized from excess carbon fixed during the light period and subsequently mobilized to sustain metabolism during the night or under nutrient‐deprived conditions (Hartman et al., 2023).

Starch is the primary carbon reserve in plants. It consists of two distinct α‐glucans: amylose, a mostly linear polymer of α‐1,4‐linked glucose units, and amylopectin, which contains α‐1,4‐linked chains with α‐1,6‐linked branch points. The regular spacing of branches in amylopectin allows adjacent chains to form double helices that pack into ordered, crystalline lamellae. These alternate with amorphous regions, resulting in a semi‐crystalline structure that underlies the limited solubility of starch (MacNeill et al., 2017). In green tissues, starch accumulates in chloroplasts during the day using intermediates of the Calvin–Benson–Bassham (CBB) cycle. At night, starch is broken down within the chloroplast into maltose and glucose, which are exported to the cytosol to fuel cellular metabolism during the dark phase (Stitt et al., 2010).

In contrast, cyanobacteria—the only prokaryotes capable of oxygenic photosynthesis—generally store carbon in the form of glycogen (Zilliges, 2014), although several unicellular diazotrophic species accumulate insoluble starch‐like polysaccharides (Kuroki et al., 2025). Structurally, glycogen differs from starch in having shorter α‐1,4‐linked chains and a higher frequency of α‐1,6 branches, resulting in highly branched, soluble granules (Preiss, 1984). Like starch, glycogen is synthesized during the light period from photosynthetically fixed carbon. Additionally, unicellular cyanobacteria produce glycogen as a response to nutrient limitation (Klotz & Forchhammer, 2017). During dark periods and during the recovery from nutrient‐limiting conditions, glycogen degradation releases glucose‐1‐phosphate, which is metabolized via the oxidative pentose phosphate (OPP) pathway to support heterotrophic growth (Doello et al., 2018).

Given the central role of these polymers as a source of energy during day/night cycles and environmental stresses, the precise regulation of starch and glycogen degradation is essential for the survival of photosynthetic organisms. Tight control of the activity of glycogen and starch catabolic enzymes is of crucial importance to ensure that stored carbon is used efficiently and in accordance with cellular needs (Doello et al., 2022; Ribeiro et al., 2022). In recent years, structural studies of starch‐ and glycogen‐degrading enzymes have provided valuable insights into their catalytic mechanisms and modes of regulation.

**Figure 1:**
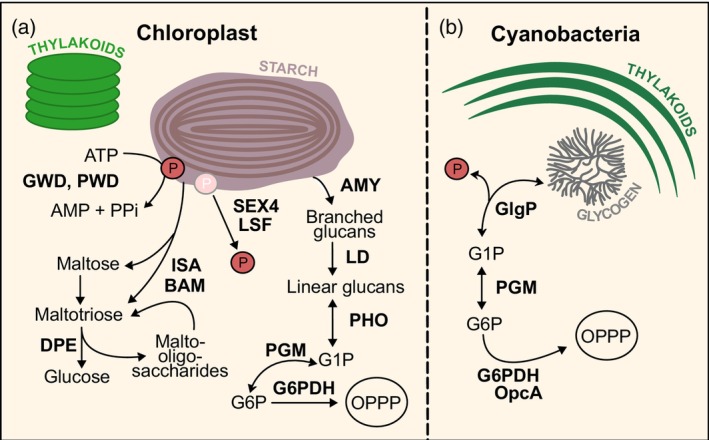
Scheme of α‐glucan catabolism in photosynthetic organisms. (a) Starch degradation in chloroplasts. AMY, α‐amylase; BAM, β‐amylase; DPE, disproportionating enzyme; G1P, glucose‐1‐phosphate; G6P, glucose‐6‐phosphate; G6PDH, glucose‐6‐phosphate dehydrogenase; GWD, glucan, water dikinase; ISA, isoamylase; LD, limit dextrinase; LSF, Like SEX Four; PGM, phosphoglucomutase; PHO, starch phosphorylase; PWD, phosphoglucan, water dikinase; SEX4, phosphoglucan phosphatase. (b) Glycogen degradation in cyanobacteria. GlgP, glycogen phosphorylase; OpcA, oxidative pentose phosphate cycle protein.

**Figure 2:**
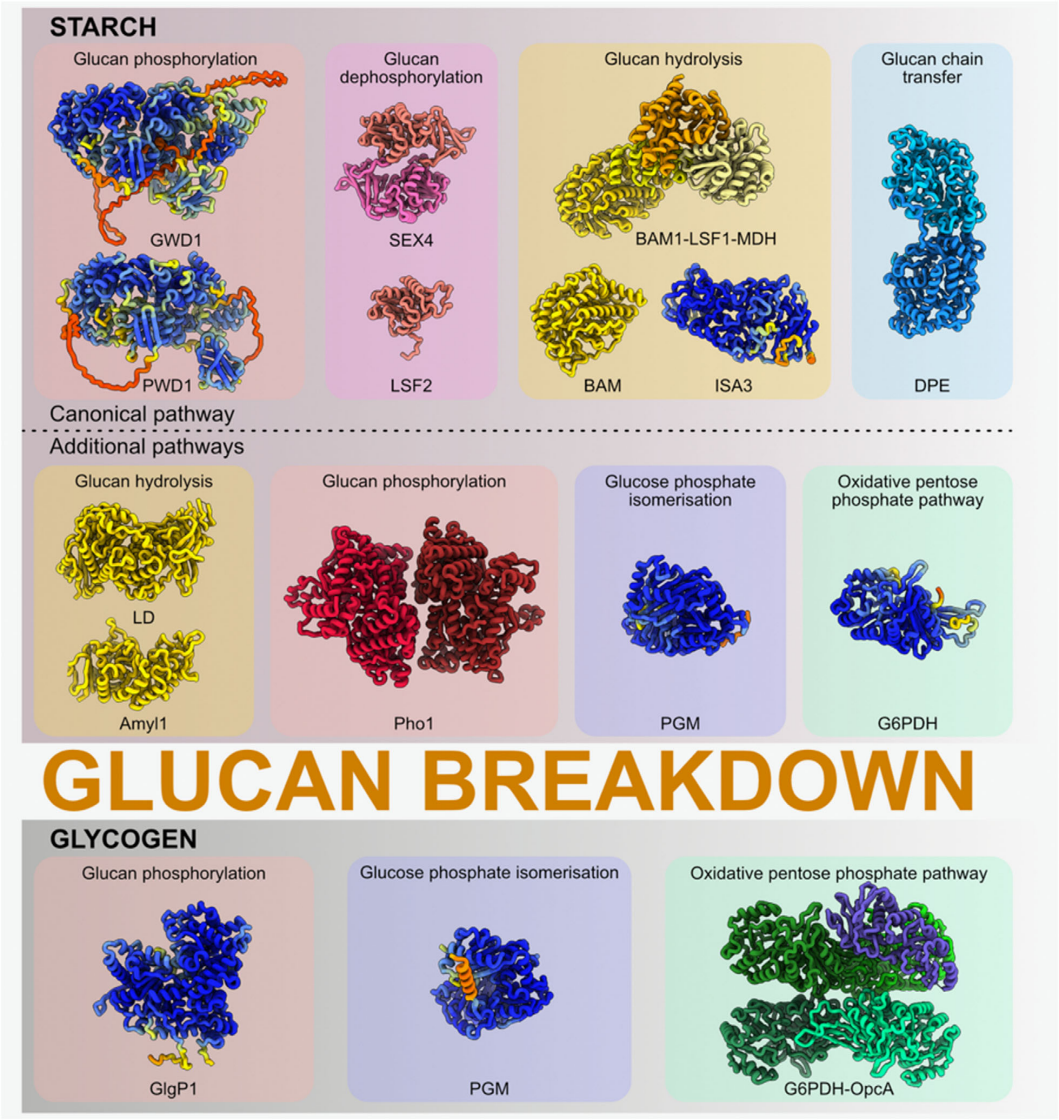
Structures of enzymes involved in starch and glycogen catabolism. Experementally determined structures and AlphaFold (AF) models of enzymes involved in starch breakdown (top) and glycogen breakdown (bottom) are shown. AF models are colored by the Local Distance Difference Test (pLDDT) confidence measure (blue, pLDDT = 100, high model accuracy expected; red, pLDDT = 0, low model accuracy, may be disordered). Enzymes are grouped by function in respectively colored boxes. Light pink boxes, glucan phosphorylation enzymes GWD1 (AF‐Q9SAC6), PWD1 (AF‐Q6ZY51), Pho1 (PDB:5LR8), GlgP1 (AF‐P73511); pink box, glucan dephosphorylation enzymes LSF2 (PDB:4KYR) and SEX4 (PDB:3NME); sand boxes, glucan hydrolysis enzymes BAM (PDB:1BTC), BAM1‐LSF‐MDH complex (PDB:8J5D), Amyl1 (PDB:3WN6), LD (PDB:2Y4S), ISA3 (AF‐Q9M0S5); light blue box, glucan chain transfer enzyme DPE (PDB:5CPQ); blue boxes, glucose phosphate isomerization enzymes plant (starch) and cyanobacterial (glycogen) PGM (plant—AF‐Q9SCY0, cyanobacterial—AF‐P74643); green boxes, oxidative pentose phosphate pathway enzymes plant G6PDH (AF‐Q43727) (starch) and cyanobacterial complex G6PDH‐OpcA (PDB:9EMM) (glycogen). Multiprotein complexes or oligomers are colored by subunits.

This review focuses on recent structural and mechanistic advances in our understanding of α‐glucan catabolism in photosynthetic organisms (Figure 1). By integrating structural data with biochemical observations, we aim to summarize how recent findings in enzyme architecture reveal the regulatory mechanisms involved in glycogen and starch degradation in cyanobacteria and plant chloroplasts (Figure 2). In particular, we will examine structural insights into the enzymes catalyzing the breakdown of starch and glycogen into mono‐ and disaccharides, and into glucose‐6‐phosphate dehydrogenase (G6PDH), a key enzyme linking α‐glucan degradation to the OPP pathway.

## STARCH DEGRADATION IN CHLOROPLASTS

### Canonical pathway

The outer surface of starch granules must be solubilized to facilitate access and degradation by glycolytic enzymes (Figure 1a, left, Figure 2, top row). Local starch solubilization requires initial ATP‐dependent phosphorylation of the polymer granule surface by glucan, water dikinase (GWD) at the C6 positions of glucosyl residues and by phosphoglucan, water dikinase (PWD) at the C3 positions to initiate the solubilization (Mahlow et al., 2016; Ritte et al., 2006). So far, the mechanistic understanding of GWD and PWD is based on biochemical and mutagenesis data, as well as their homology to other enzymes, while high‐resolution structural information is missing. GWD was discovered as a protein able to bind and phosphorylate starch granules in the late 1990s (Lorberth et al., 1998). Based on the homology to pyruvate, water dikinase and pyruvate, phosphate dikinase, it has been demonstrated by mutational analysis that a conserved histidine from the phosphohistidine domain of GWD—His992 in the potato (*Solanum tuberosum*) enzyme—is essential for the catalytic activity (Mikkelsen et al., 2004). Later, *in vitro* phosphorylation experiments followed by mass spectrometry showed that GWD introduces a single phosphate group in close vicinity to the branching points (Compart et al., 2023). GWD was shown to be redox regulated by thioredoxins, linking starch degradation to the dark/light cycle (Mikkelsen et al., 2005). The regulation occurs through reversible oxidation and formation of an intramolecular disulfide bridge near the catalytic histidine of GWD, resulting in enzyme inactivation. Notably, the oxidized and inactive form constitutes the predominant fraction of GWD attached to starch granules in dark‐adapted plants, suggesting that during the early stages of starch degradation in the dark, oxidized GWD first binds to starch and may subsequently be activated by thioredoxins (Mikkelsen et al., 2005). In addition to redox regulation, GWD is controlled through phosphorylation by the target of rapamycin (TOR) kinase, a conserved eukaryotic enzyme central to the control of various metabolic processes (Komiya et al., 2025). In turn, PWD, which preferentially phosphorylates the C3 position of glucose units, presumably shares a similar mechanism with GWD but requires starch prephosphorylation by GWD (Baunsgaard et al., 2005; Kötting et al., 2005). Due to the absence of high‐resolution structures of GWD and PWD, future structural studies will be essential to dissect the substrate binding using short glucan fragments of varying length and structure, and to elucidate the conformational changes underlying catalysis. Another key open mechanistic question concerns how these enzymes recognize and bind the glucan granule surface, which will require *in vitro* and *in situ* structural studies combined with biophysical approaches and targeted mutagenesis.

After phosphorylation by GWD and PWD, the partly solubilized starch granule surface must be dephosphorylated to allow access for the hydrolytic enzymes. Starch dephosphorylation is carried out by several glucan phosphatases (Gentry et al., 2016; Meekins et al., 2016), including Starch EXcess4 (SEX4) and Like SEX Four (LSF1 and 2). Crystal structures revealed that SEX4 is a 42 kDa protein containing a dual‐specificity phosphatase (DSP) domain and a carbohydrate binding module (CBM). Interconnected DSP and CBM are both involved in glucan binding, and DSP harbors the active site with a catalytically reactive cysteine residue (Mak et al., 2021; Meekins et al., 2014). In addition, the DSP domain of SEX4 contains two phenylalanine residues (Phe235 and Phe140 in *Arabidopsis thaliana*) that shape the active site topology, positioning the glucan substrate and conferring preferential specificity for C6 dephosphorylation of the starch glucose moiety while retaining residual C3‐directed activity (Meekins et al., 2014). Indeed, replacing these phenylalanine residues in SEX4 with their counterparts from C3‐specific LSF2 shifts substrate specificity of SEX4 toward C3 (Meekins et al., 2014), highlighting the potential for engineering starch phosphorylation in plants. Similarly to GWD, SEX4 is redox regulated, with its catalytic cysteine forming a disulfide bridge with another cysteine, rendering SEX4 reversibly inactive upon oxidation (Silver et al., 2013).

LSF1 and 2 were described as additional phosphatases that remove phosphate groups from starch polymers (Comparot‐Moss et al., 2010; Santelia et al., 2011). Structures of LSF2, with and without phospho‐glucan product, revealed that LSF2 lacks the CBM domain and instead binds glucan through its phosphatase (DSP) domain, which specifically dephosphorylates glucose at the C3 position (Meekins et al., 2013). In contrast to SEX4, the substrate‐binding site of the LSF2 DSP domain contains glycine and tryptophan residues (G230 and W136 in *Arabidopsis thaliana*) that facilitate positioning of the substrate for C3‐specific dephosphorylation (Meekins et al., 2013; Meekins et al., 2014). Interestingly, several additional glucan‐binding sites were identified on the surface of LSF2, which were hypothesized to functionally substitute for a CBM domain and were shown to be critical for the LSF2 activity. A recent study combining crosslinking mass spectrometry and cryogenic electron microscopy (cryo‐EM) demonstrated that in *Arabidopsis thaliana*, LSF1 forms a complex with NAD‐dependent malate dehydrogenase (MDH) and β‐amylase 1 (BAM1) resembling a starch‐degrading multiprotein complex (Liu et al., 2023). Although the full‐length structure of LSF1 remains unresolved, this study shows that its unique PDZ and BAM1/MDH interaction domains, together with MDH acting as a chaperone, form a platform for BAM1, thereby facilitating starch degradation.

Consequent starch granule phosphorylation and dephosphorylation is followed by the attack of hydrolytic enzymes, including the exoamylases (BAMs) and the debranching enzymes (Smith & Zeeman, 2020; Streb & Zeeman, 2012). BAMs hydrolyze the α‐1,4 glucosidic bonds resulting in the release of maltose. Crystal structures of different BAM proteins in both substrate‐free and substrate‐bound states (Cheong et al., 1995; Kang et al., 2004; Mikami et al., 1993; Mikami et al., 1994; Vajravijayan et al., 2018) have revealed that BAMs possess a characteristic (β/α)_8_‐barrel domain with a deep cavity containing two catalytic glutamate residues (Vajravijayan et al., 2018). These conserved glutamate residues (Glu186 and Glu380 in the soybean BAM5 enzyme) are located below and above the bound sugar substrate and function as general acid and general base catalysts (Kang et al., 2004). BAMs are active as monomers but can form tetrameric complexes for stabilization (Cheong et al., 1995). Various BAM isoforms are present in plants, including cytosolic (BAM5), nuclear (BAM7 and BAM8) and plastidic (BAM1‐4, BAM6, and BAM9) (Monroe & Storm, 2018). Plastidic BAM isoforms differ by their expression levels in various plant tissues and in their mode of regulation. BAM1, BAM2, BAM3, and BAM6 are catalytically active, while BAM4 and BAM9 lack one of the catalytic glutamate residues (Monroe & Storm, 2018). Even though BAM4 and BAM9 are inactive *in vitro* (Fulton et al., 2008; Li et al., 2009; Monroe & Storm, 2018), loss of BAM4 and BAM9 leads to starch accumulation in plants (Fulton et al., 2008), BAM4 binds amylose and amylopectin (Li et al., 2009), and BAM9 interacts with BAM1 and starch granules (Hou et al., 2017). These data collectively suggest that BAM4 and BAM9 may play regulatory roles in starch degradation, potentially being preferentially localized to specific plant tissues, by interacting with glucan substrates or proteins, such as other BAMs or additional starch‐catabolizing enzymes, or they may act on a yet‐to‐be‐identified substrate. Interestingly, BAM1 has a unique Cys32 involved in an inhibitory disulfide bond and is redox regulated by light via thioredoxin f (Sparla et al., 2006; Valerio et al., 2011). In contrast to plastidic BAMs, nuclear BAM7 and BAM8 are catalytically inactive *in vitro* and function as transcription factors. On the other hand, cytosolic BAM5 is active and has been suggested to play a regulatory role by controlling the accumulation of polysaccharides in plant tissues or by acting on shorter oligosaccharide substrates (Monroe & Storm, 2018).

Together with BAMs, starch hydrolysis is catalyzed by the debranching enzymes that include Isoamylase 1 (ISA1), ISA2, ISA3, and limit dextrinase (LD, see below) and break α‐1,6‐branch points in starch (Smith & Zeeman, 2020). Whereas ISA1 and ISA2 have been shown to participate in starch biosynthesis, ISA3 and LD are involved in starch breakdown (Streb et al., 2009). While the structure of ISA3 remains to be determined, recent X‐ray crystallography and cryo‐EM studies have revealed the architectures of the ISA1 and ISA2 enzymes in both substrate‐free and product‐bound states (Fan et al., 2025; Sim et al., 2014). ISA1 and ISA2 both contain a catalytic (β/α)‐barrel domain flanked by N‐terminal and C‐terminal domains. In *Chlamydomonas* and rice, ISA1 forms an elongated homodimer (Sim et al., 2014). The rice ISA1 enzyme, however, can also form a heterocomplex with ISA2, in which ISA2 predominantly attaches to one of the ISA1 monomers of the extended dimer and increases the debranching activity of ISA1 (Fan et al., 2025). ISA1 contains a substrate‐binding cleft within the catalytic domain, where a maltooligosachharide binds and is stabilized by a network of hydrogen bonds provided by the side chains and backbone of the cleft residues, and the catalytic aspartate residue (Asp452 in *Chlamydomonas* and Asp432 in rice) (Sim et al., 2014). In addition to the catalytic binding site, ISA1 possesses two surface (secondary) binding sites, one located at the reducing end of the catalytic cavity and another at the interface between the catalytic and C‐terminal domains. The slight curvature of these binding sites may facilitate the binding of ISA1 to the crystalline amylopectin (Sim et al., 2014). In contrast to ISA1, ISA2 has been shown to bind only four glucose molecules in its C‐terminal segment (Fan et al., 2025).

The catalytic activities of BAMs and ISA3 result in the release of maltose and maltotriose, with maltotriose subsequently degraded by the disproportionating enzyme (DPE1 or D‐enzyme). DPE1 performs transglycosylation by converting two maltotrioses into a glucose and a maltopentaose molecule, the latter of which can be further degraded by BAMs. The recent X‐ray structures of DPE1 from *Arabidopsis* and potato, in both substrate‐free and substrate‐bound states, revealed the overall architecture of the enzyme and suggested the mechanism of the catalysis (Imamura et al., 2020; O'Neill et al., 2015). DPE1 resembles an elongated dimer with each monomer possessing a conserved (β/α)₈‐barrel domain (A) ringed by three additional subdomains (B1–B3). The subdomains B1–B3 form a deep substrate cavity located perpendicularly to the domain A barrel axis. The catalytic residues (Asp373, Glu420, and Asp473 in *Arabidopsis* and Asp321, Glu368, and Asp421 in potato) are situated at the bottom of the cavity, separating the donor and acceptor binding sites. Substrate‐bound structures of DPE1 revealed that the enzyme does not undergo large‐scale conformational changes compared with the apo state. In contrast, only local rearrangements of several residues are observed, with Asp321 playing an essential role in substrate binding and in the formation of a covalent glycosyl‐enzyme intermediate (Imamura et al., 2020).

### Additional starch catabolic pathways

Beyond the canonical pathway of starch degradation mediated by β‐amylases and debranching enzymes, chloroplasts also harbor alternative routes involving α‐amylases (AMY), LDs, and starch phosphorylases (PHO) (Figure 1a, right, Figure 2, middle row). These enzymes broaden the mechanistic repertoire of starch breakdown, and their activity is subject to regulation through distinctive structural features.

α‐Amylases catalyze the hydrolysis of α‐1,4 glycoside linkages to yield maltose and maltodextrin products. The structural aspects of the regulation of α‐amylases were reviewed earlier (Møller & Svensson, 2016). In the Carbohydrate‐Active enZYmes (CAZy) database, α‐amylases are assigned to three glycoside hydrolase (GH) families—GH‐13, GH‐57, and GH‐119—with most plant α‐amylases belonging to GH‐13. Members of this family share a conserved tripartite architecture consisting of a catalytic (β/α)₈‐barrel domain (domain A), a long‐loop domain inserted within domain A (domain B), and a C‐terminal β‐sheet domain (domain C). The active site, located in domain A, is defined by three conserved residues (Asp‐Glu‐Asp) and stabilized by a variable number of bound calcium ions. The most recent plant α‐amylase solved structure is that of AmyI‐1 from rice (*Oryza sativa*) (Ochiai et al., 2014). In this enzyme, three calcium ions stabilize the structure, and the catalytic residues correspond to Asp203, Glu228, and Asp314, with Glu228 being the residue that initiates the acid–base catalysis. Many α‐amylases also contain secondary carbohydrate binding sites on the molecular surface. Rice AmyI‐1 contains two surface binding sites, located in domain A and domain B, with residues Trp301, Trp302, and Tyr403 playing a key role in carbohydrate binding. A distinctive regulatory feature of plant α‐amylases is the presence of an N‐linked glycosylation consensus site. In rice AmyI‐1, N‐linked glycosylation enhances thermostability and is required for chloroplast targeting (Ochiai et al., 2014).

LDs hydrolyze the α‐1,6‐glucosidic linkages of branched maltooligosaccharides produced by α‐amylases. LDs also belong to the CAZy family GH‐13 and share the domain architecture with α‐amylases but differ in the type of glycosidic bond they can hydrolyze and in their substrate specificity. Several crystal structures of barley LD have been reported in complex with its substrate and inhibitors, providing detailed insights into the structural basis of their activity and regulation (Møller, Vester‐Christensen, et al., 2015; Møller, Windahl, et al., 2015; Vester‐Christensen et al., 2010). The length of the main chain can be up to five glucose units long, although two is the optimal number of glucose units that can be accommodated at the non‐reducing side of the main chain after the α‐1,6‐glucosidic linkage. Residue Met440 and a dynamic loop containing Phe620‐Asp621 are involved in substrate specificity and are thought to impede the binding of substrates with a longer main chain (Møller, Vester‐Christensen, et al., 2015; Vester‐Christensen et al., 2010). A key structural determinant of LD activity is the arrangement of aromatic residues flanking the catalytic cleft. In barley LD, Trp512 and Phe553 play central roles in positioning the α‐1,6‐glucosidic linkage for hydrolysis. Trp512 is conserved across members of the GH13 family, whereas Phe553 is specifically found in enzymes capable of cleaving α‐1,6‐linkages. Interaction with these residues excludes non‐branched polysaccharides from reaching the catalytic nucleophile and acid–base residues, while it orients branched substrates so that the α‐1,6 bond is optimally placed for cleavage, therefore avoiding dual α‐1,6‐ and α‐1,4‐hydrolytic activity (Møller, Windahl, et al., 2015).

PHO catalyzes the reversible conversion of starch and inorganic phosphate into glucose‐1‐phosphate, thus being involved in starch synthesis and degradation (Rathore et al., 2009). PHO enzymes belong to the CAZy GT35 family of glycosyltransferases and exist in two isoforms in plants: the plastidic PHO1 and the cytosolic PHO2 (Shoaib et al., 2021). While they share significant sequence similarity, the two isoforms differ in size, localization, kinetic properties, and regulatory features. PHO1 represents the main isoenzyme, accounting for more than 95% of the total phosphorylase activity in several plants (Hwang et al., 2020). PHO enzymes are composed of four domains: an N‐terminal domain, a regulatory domain, the L78 (also called L80) domain, and a C‐terminal catalytic domain. Whereas the regulatory and catalytic domains are highly conserved, the N‐terminal and L78 domains vary substantially among species. The binding site for the essential pyridoxal 5′‐phosphate (PLP) cofactor is located in the C‐terminal catalytic site. The L78 domain is only present in PHO1 and not in PHO2 isoforms. This region, varying in length between 50 and 82 amino acids among different plant species, constitutes a key structural regulatory element in PHO1 (Hwang et al., 2020).

The first structure of a plastidic plant starch phosphorylase was solved by (Cuesta‐Seijo et al., 2017), who reported the crystal structure of PHO1 from barley (*Hordeum vulgare*). Barley PHO1 forms a homodimer, a common but not universal feature of PHO proteins. Structural and functional studies in barley and potato (*Solanum tuberosum*) PHO1 revealed that L78 acts as a regulatory loop: its presence hinders substrate access, while proteolytic removal increases substrate affinity and catalytic activity (Cuesta‐Seijo et al., 2017; Koulas et al., 2024). In potato PHO1, removal of the L78 region produces an enzyme that is 1.5 times more active than the non‐proteolyzed enzyme and displays stronger substrate affinity (Koulas et al., 2024). By contrast, in the rice (*Oryza sativa*) PHO1 removal of the L78 segment does not affect catalytic efficiency or substrate affinity but contributes to heat stability (Hwang et al., 2016). The regulatory function of L78 is further linked to a PEST motif, a sequence rich in proline (P), glutamic acid (E), serine (S), and threonine (T) that serves as a signal for proteolytic degradation. In several PHO1 enzymes, the L78 domain contains a PEST motif at its N‐terminal side, coupling proteolysis to enzymatic activation. However, PEST sequences are absent from L78 regions of some species, such as bonnet pepper (*Capsicum chinense*) and barrel clover (*Medicago truncatula*), suggesting that proteolysis‐dependent activation is not a universal regulatory mechanism in higher plants (Hwang et al., 2020). Although further regulatory mechanisms have not been identified for PHO1 in bonnet pepper and barrel clover, studies in other organisms have shown that PHO1 activity is also modulated via phosphorylation and protein–protein interactions.

Phosphorylation of the L78 domain has been shown in some plants, including maize and *Ipomoea batatas* (sweet potato). This post‐translational modification makes PHO1 more susceptible to proteolytic cleavage but does not affect its kinetic properties (Hwang et al., 2020). Biochemical studies in rice endosperm demonstrated that PHO1 forms a stable 1:1 complex with the disproportionating enzyme DPE1, as shown by co‐immunoprecipitation, blue‐native PAGE, and co‐elution experiments (Hwang et al., 2016). Complex formation does not require the L78 domain. Functional assays revealed that the PHO1–DPE1 complex displays enhanced substrate affinity and expanded glucan utilization compared with the individual enzymes, suggesting a synergistic role in starch degradation. Although crystal structures of the individual proteins are available—PHO1 from barley and potato (Cuesta‐Seijo et al., 2017; Koulas et al., 2024), and DPE1 from *Arabidopsis thaliana* (O'Neill et al., 2015)—a high‐resolution structure of the PHO1–DPE1 complex has not yet been determined. Thus, the molecular interface and the structural basis of their synergistic regulation remain open questions.

## GLYCOGEN CATABOLISM IN CYANOBACTERIA

In cyanobacteria (Figures 1b and 2, bottom row), the functional homolog of plastidic starch phosphorylase (PHO1) is glycogen phosphorylase (GlgP), which also belongs to the CAZy GT35 family. Although no cyanobacterial GlgP structures have yet been solved, sequence alignments across diverse organisms reveal strong structural similarity with plant PHO1. Like PHO1, cyanobacterial GlgPs function as homodimers and require pyridoxal 5′‐phosphate (PLP) as a cofactor (Koulas et al., 2024). The model unicellular cyanobacterium *Synechocystis* sp. PCC 6803 encodes two glycogen phosphorylase isoforms, GlgP1 and GlgP2, which are differentially regulated depending on environmental conditions. While plants partition PHO isoforms across tissues, cyanobacteria instead exploit isoform specialization to adapt to different stresses: GlgP2 is critical for survival under prolonged nutrient limitation, whereas GlgP1 is required during heat stress (Neumann et al., 2025).

In mammals, GlgP is regulated by phosphorylation and the levels of adenosine monophosphate. Such a regulation has not been reported in cyanobacteria. A distinctive regulatory feature of cyanobacterial GlgPs is the presence of a C‐terminal regulatory peptide, which contrasts with the internal L78 loop of PHO1. Sequence alignment of bacterial GlgPs showed that such a terminal extension is a unique feature of the cyanobacterial enzymes. Structural models generated with AlphaFold predict this C‐terminal region as a flexible loop. Although *Synechocystis'* GlgP1 and GlgP2 share similar structures of their catalytic site and catalytic capacity, they differ in the composition of their C‐terminal domain. In GlgP1, the peptide contains two cysteine residues capable of forming a disulfide bond, thereby rendering the enzyme sensitive to the redox state of the cell. Indeed, GlgP1 activity is modulated by the thioredoxin system, which links glycogen breakdown to the photosynthetic reduction of ferredoxin. In contrast, the C‐terminal peptide of GlgP2 lacks cysteine residues, and its role in regulating enzymatic activity remains unresolved (Neumann et al., 2025). These observations suggest the C‐terminal regulatory peptide might represent the regulatory mechanism that accounts for the different functional specialization of GlgP1 and GlgP2 in cyanobacteria.

Phosphoglucomutase (PGM) catalyzes the reversible interconversion of glucose‐1‐phosphate and glucose‐6‐phosphate, thereby linking glucan degradation with central carbon metabolism (Doello & Forchhammer, 2023). PGMs are highly conserved enzymes, and although no crystal structures have yet been determined for photosynthetic organisms, structural studies of mammalian, bacterial, and archaeal enzymes show a characteristic four‐domain fold (Liu et al., 1997; Mehra‐Chaudhary et al., 2011; Müller et al., 2002; Naz et al., 2025; Regni et al., 2004). The catalytic serine residue located in domain one becomes transiently phosphorylated, forming a phospho‐enzyme intermediate essential for catalysis. The reaction mechanism proceeds through two phosphoryl transfer steps. First, the catalytic serine donates its phosphate group to glucose‐1‐phosphate, generating the reaction intermediate glucose‐1,6‐bisphosphate. The intermediate then undergoes a 180° reorientation within the active site, after which the second phosphoryl transfer occurs, restoring the phosphate on the catalytic serine and releasing glucose‐6‐phosphate. Domain movements accompany this substrate “flip,” allowing controlled access to and positioning within the active site, and underpin the intrinsic reversibility of the reaction (Regni et al., 2004). In cyanobacteria, PGMs contain an additional regulatory phosphorylation site. In *Synechocystis* sp. PCC 6803, phosphorylation of Ser47—located in a peripheral region involved in substrate binding and active site closure—renders the enzyme inactive. This phosphorylation site is not found in the plant enzyme but is conserved in mammals (Doello et al., 2022). This differential regulation in cyanobacterial and plant PGM might derive from the evolutionary shift from glycogen‐ to starch‐based carbon storage.

G6PDH utilizes glucose‐6‐phosphate produced by PGM activity from glucose‐1‐phosphate derived from storage glucans. G6PDH produces 6‐phosphogluconolactone catalyzing the first and rate‐limiting step of the OPP pathway, which is an essential glycolytic shunt of the CBB cycle in autotrophic prokaryotes and is essential for nicotinamide adenine dinucleotide phosphate (NADPH) production, synthesis of nucleotides and balancing the cellular redox state (Jiang et al., 2022; Makowka et al., 2020; Stanton, 2012). G6PDH usually forms homodimers or homotetramers with each monomer consisting of a smaller N‐terminal coenzyme β–α–β dinucleotide‐binding domain connected to a larger C‐terminal β + α domain possessing a large curved 9‐stranded β‐sheet (Au et al., 2000; Doello et al., 2024; Rowland et al., 1994; Wei et al., 2022). The G6PDH active site, located at the interface between the C‐ and N‐terminal domains of the enzyme, is characterized by the conserved sequence DHYLGK and binds the substrate molecules of glucose‐6‐phosphate and catalytic NADP^+^. In contrast, the mechanisms of G6PDH allosteric regulation vary among different organisms. Whereas the human enzyme is activated through the binding of an additional, structural NADP^+^ molecule to the C‐terminal domain β‐sheet (Au et al., 2000; Wei et al., 2022), G6PDH from photoautotrophs is regulated via redox modifications (Doello et al., 2024; Hagen & Meeks, 2001; Née et al., 2009; Née et al., 2023). Plastidic G6PDH contains two surface‐exposed cysteine residues in the N‐terminal domain that are proposed to form a disulfide bond upon interaction with thioredoxin f, thereby activating the enzyme (Née et al., 2009; Wenderoth et al., 1997). However, structural evidence for this mechanism is still lacking. Interestingly, the cyanobacterial enzyme lacks these cysteine residues; however, it is still redox regulated through the interaction with the redox‐sensitive protein OpcA (Hagen & Meeks, 2001). While integration of redox regulation within G6PDH in plants likely makes control of enzyme activity more efficient, more complex OpcA‐mediated regulation might be particularly beneficial in cyanobacteria since photosynthesis and central carbon metabolism occur in a single, less compartmentalized cellular space. The structure of the G6PDH‐OpcA complex from *Synechocystis* sp. PCC 6803 revealed that a single OpcA molecule binds to the C‐terminal domain of one monomer within the G6PDH tetramer, analogous to the structural NADP^+^ molecule observed in the human enzyme. The C‐terminal domain of OpcA that forms an interface with G6PDH contains a disulfide bond between Cys412‐Cys416. According to the proposed mechanism, redox control of G6PDH activity occurs indirectly through the formation of a disulfide linkage in the bound OpcA protein, which induces structural rearrangements that allosterically regulate G6PDH (Doello et al., 2024).

## CONCLUSION AND OUTLOOK

Starch and glycogen are the main polyglucans used for energy storage by living organisms. Glucan degradation pathways have been studied for decades, and the determination of 3D structures of the involved enzymes has shed light on their catalytic mechanisms. However, the structures of several enzymes catalyzing key steps of glucan degradation, such as GWD1, PWD1, and ISA3, have yet to be determined, leaving their mechanistic understanding to structural modeling and homology‐based functional analyses. Advances in single particle cryo‐EM have not only enabled the structural analysis of large and flexible proteins and protein complexes but also made this technique more accessible to many laboratories, holding great potential for future structural studies of glucan degradation. This trend is exemplified by the recently characterized structures of multiprotein complexes such as ISA1‐ISA2 (Fan et al., 2025), BAM1‐LSF1‐MDH (Liu et al., 2023), and G6PDH‐OpcA (Doello et al., 2024), which had resisted crystallographic analysis. Moreover, recent studies using electron cryotomography (cryo‐ET) (Beck & Baumeister, 2016) have uncovered the *in situ* organization of starch structures in the *Chlamydomonas* chloroplast (Engel et al., 2015), demonstrating the potential of cryo‐ET for future structural analysis of starch metabolism. However, cryo‐ET analysis of starch granules remains challenging due to their semi‐crystalline amorphous ultrastructure (Brust et al., 2020) and their sensitivity to radiation damage (Engel et al., 2015). Future cryo‐ET studies of glucan granules will require complex workflows that combine multiple techniques, including focused ion beam milling (Klumpe & Plitzko, 2025) to thin samples and correlative light and electron microscopy (Pierson et al., 2024) to localize specific proteins of interest. Nevertheless, such *in situ* studies will enable quantification and investigation of the intracellular spatial organization, morphology, and formation or degradation of glucan granules—similarly to a recent study of polyhydroxybutyrate granules in *Caulobacter* (De Koning et al., 2023)—as well as the analysis of protein assemblies at the granule surface that may be involved in glucan metabolism. Thus, despite recent progress in understanding the mechanisms of glucan catabolism, future studies integrating functional assays with a combination of cryo‐EM and cryo‐ET (Nogales & Mahamid, 2024) will be necessary to address remaining gaps in our knowledge of the molecular mechanisms and spatiotemporal regulation of glucan‐degrading enzymes in photosynthetic organisms.

## Data Availability

No data was used for the research described in the article.
